# A novel treatment strategy of new onset atrial fibrillation after cardiac surgery: an observational prospective study

**DOI:** 10.1186/1749-8090-9-83

**Published:** 2014-05-12

**Authors:** Mohamed Zeriouh, Anton Sabashnikov, Yeong-Hoon Choi, Javid Fatullayev, Hannes Reuter, Aron-Frederik Popov, Georg Langebartels, Lucas Kimmig, Parwis B Rahmanian, Thorsten Wittwer, Klaus Neef, Jens Wippermann, Thorsten Wahlers

**Affiliations:** 1 Department of Cardiothoracic Surgery, Heart Center of the University of Cologne, Cologne, Germany; 2 Department of Cardiology, Pneumology and Angiology, Heart Center of the University of Cologne, Cologne, Germany; 3 Center for Molecular Medicine Cologne, University of Cologne, Cologne, Germany; 4 Department of Thoracic and Cardiovascular Surgery, University Hospital of Goettingen, Goettingen, Germany

**Keywords:** Vernakalant, Flecainide, Recent onset atrial fibrillation post cardiac surgery, Electrical cardioversion, Antiarrhythmic drugs

## Abstract

**Objective:**

The aim of this prospective observational study was to evaluate the efficiency of a new escalating treatment strategy with vernakalant, flecainide and electrical cardioversion (EC) in patients with new onset atrial fibrillation (AF) after cardiac surgery.

**Material and methods:**

24 patients with new onset AF after aortic valve surgery, coronary artery bypass surgery or combined procedures were evaluated in this study. Additional including criteria were age between 18 and 80, duration of AF less than four days, body weight less than 100 kg and no previous treatment with class I or III antiarrhythmic drugs. Exclusion criteria were poor left ventricular ejection fraction (LVEF < 40%) and history of myocardial infarction within 30 days. The patients were divided into converters and non-converters according to their response to combination treatment with vernakalant and flecainide, and the groups were compared.

**Results:**

The mean age of the population was 69.6 ± 6.3 years and 26.1% of patients were female. There were no statistically significant differences between the two groups in terms of height, weight, gender distribution, comorbidities, preoperative medication, left ventricular function and left atrium diameter. Interventricular septum (IVS) in the non-converted group was significantly thicker compared to the converted group: 14.0 ± 1.00 vs. 10.40 ± 2.59 mm (p = 0.036). While 14 patients (60.9%) were successfully converted into stable sinus rhythm by pharmacological treatment with vernakalant and flecainide, 9 patients (39.1%, non-converted group) remained in AF. However, seven of them could be converted after additional EC.

**Conclusion:**

The combination of vernakalant and flecainide improves the conversion rate into a stable sinus rhythm in postcardiotomy patients with new onset AF compared to single drug therapy. Furthermore it might be an excellent precondition for successful EC in patients who are not converted after using both antiarrhtythmic drugs. Furthermore, left ventricular hypertrophy might be a potential negative predictor of successful pharmacological cardioversion.

## Background

Postoperative atrial fibrillation (AF) is the most common complication after cardiac surgery, which occurs in up to 74% of patients who underwent on-pump and off-pump procedures [1,2]. Furthermore, it may contribute to longer hospital stays which can be associated with deterioration in heart failure, prolonged inotropic support, use of intraaortic balloon pump, and increased risk for cerebrovascular accidents [3,4]. After more than a century of research, the etiology and pathophysiology of this arrhythmia is still not completely understood [2,5]. In terms of treatment, there are several drugs, such as digitalis, Beta-blockers, calcium channel-blockers, and pharmacological or electrical cardioversion (EC), which were shown as efficient treatment strategies of AF in patients undergoing cardiac surgery [6]. The choice of treatment in each individual case depends on a number of factors, such as age, comorbidities and clinical status of patients with AF. Also, duration and etiology of AF play a major role in the treatment choice [7].

If not approached within a short period of time, a delayed cardioversion may aggravate AF-associated symptoms and promote structural changes of the atria [8]. Therefore, it is of particular interest to achieve early cardioversion in patients with AF, especially when they suffer from distressing symptoms or seriously compromised cardiac function [1,3,9-11].

Vernakalant is an antiarrhythmic agent, which is used for rapid cardioversion of recent onset atrial fibrillation. It blocks early-activating K^+^ atrial channels and frequency-dependent atrial Na^+^ channels, prolongs atrial refractory periods and decreases atrial conduction without promoting ventricular arrhythmia [12]. Flecainide is a class Ic antiarrhythmic agent, which can be used for the prevention of ventricular and supraventricular arrhythmias [13]. Electrical cardioversion delivers a direct current synchronization with the R wave. It is usually done using external, transcutaneous electrode patches or internal cardiac electrodes [14]. All these treatment strategies have been shown as valuable options in case of postcardiotomy AF [12-14], however so far there has been no evidence regarding efficacy of stepwise escalating therapy using vernacalant, flecainide and EC after cardiac surgery.

In this study, we created a new cardioversion protocol and assessed its cardioversion efficacy in postoperative patients with recent new onset AF.

## Methods

### Patients

Between December 2011 and September 2012, 24 patients with new onset AF who underwent isolated or combined aortic valve replacement and/or coronary artery bypass surgery performed at our institution were evaluated in this observational prospective study. Inclusion criteria were: age between 18 and 80, duration of new onset AF less than 4 days, body weight less than 100 kg and no previous treatment with class I or III antiarrhythmic drugs. Preoperative exclusion criteria were: poor left ventricular ejection fraction (LVEF < 40%), NYHA class III-IV, history of myocardial infarction within 30 days before surgery, long QT syndrome (uncorrected QT interval > 440 ms), sick sinus node syndrome, higher degree atrio-ventricular block (II°, III°) as well as bradyarrhythmia absoluta. Re-do cardiac surgery patients were also excluded from this observation.

Ethical clearance for this observational prospective study was given by the institutional ethics committee of Cologne University. Therefore, according to the Ethics Committee a signed informed consent was not mandatory as both antiarrhythmic drugs used were approved in the ESC Guidelines and the risks are similar to the current standard therapy [15].

### Study protocol

**Figure 1:**
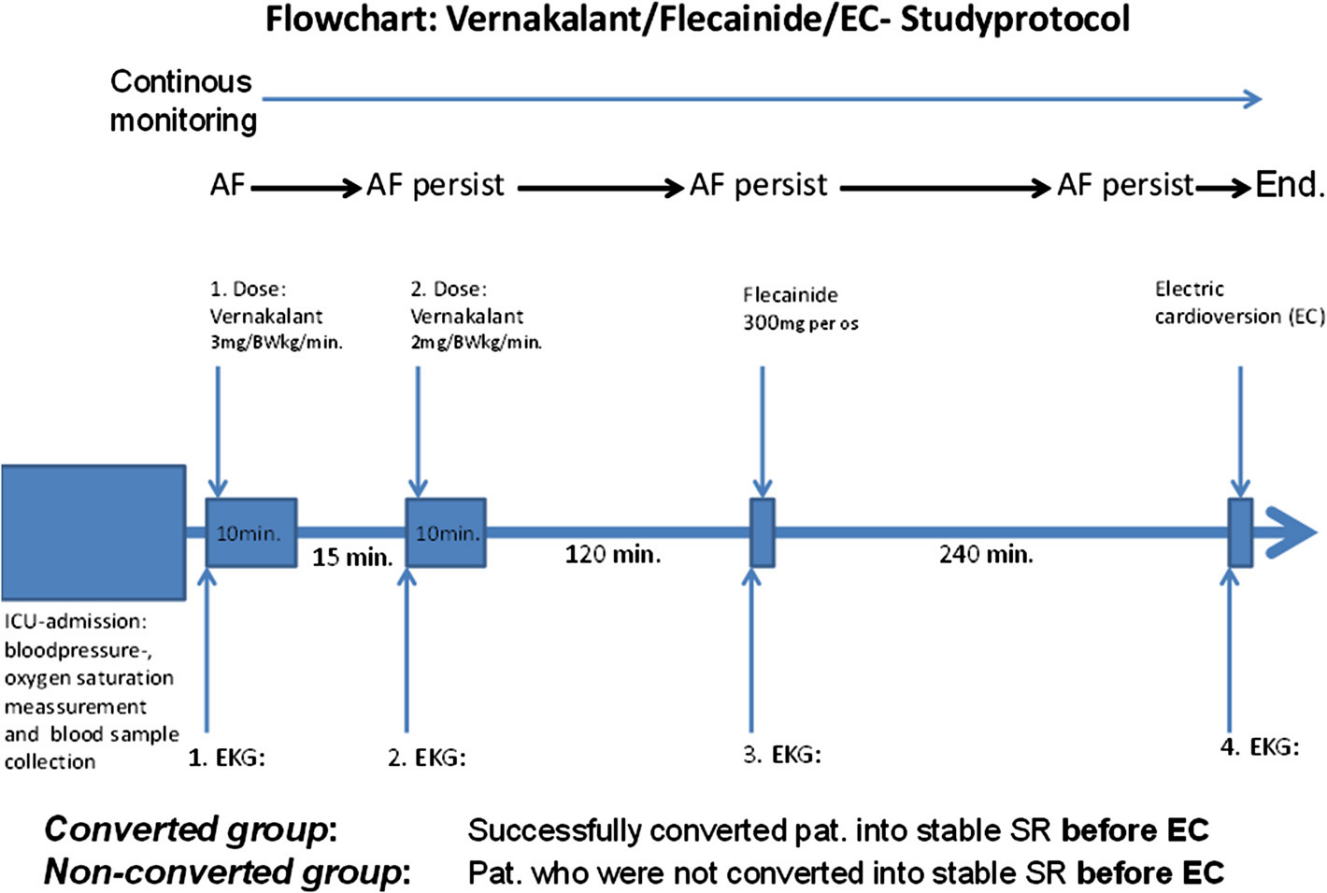
Flowchart of studyprotcol which begins with the admission to ICU with bloodpressure-, oxygen saturation measurement and blood sample collection and ends with EC.

Patients with new onset AF after a cardiac surgical procedure underwent treatment according to the protocol shown in Figure 1. In all patients who were not effectively anticoagulated for more than 48 hours after new onset AF, an elective pre-interventional trans-esophageal echocardiography was performed in order to exclude intracardiac thrombus formations before pharmacological and/or electrical cardioversion were commenced. Before the first vernakalant administration, electrolyte levels (magnesium, potassium) were checked and optimized, if indicated.

Electric cardioversion under sedation with weight adjusted etomidate was only performed in case of persistent AF after sequential pharmacological treatment with both vernakalant and flecainide. A successful cardioversion was defined as a conversion into a stable sinus rhythm within the time periods described in Figure 1.

Furthermore, according to the study protocol, all patients were divided in two groups: patients who successfully converted into a stable SR (converted group) and patients who were not converted into a SR (non-converted group) after single treatment with vernakalant or combination vernakalant/flecainide before EC. Both groups were compared regarding patients baseline preoperative, intraoperative and postoperative characteristics, past medical history, laboratory and echocardiographic parameters before treatment with the view to elaborating possible predictors of non-responders to pharmacological treatment.

### Statistical analysis

All data were analyzed using Statistical Package for Social Sciences, version 21.0 (SPSS Inc., Chicago, Illinois) and are expressed as the mean ± standard deviation (SD) in cases of normal distributed or median (interquartile range) in cases of non-normal distributed continuous variables. Variables were tested for normal distribution using the one sample Kolmogorov-Smirnov test. The Student *t* test and the Mann–Whitney *U* test were applied for comparison of continuous variables in case of normal or non-normal distribution, respectively. Chi-square and Fisher exact test were used for comparison of dichotomous variables. A p-value < 0.05 was considered to indicate statistical significance.

## Results

Tables 1, 2 and 3 represent basic demographic data, patient comorbidities, medical history, echocardiographic parameters and general clinical characteristics of all patients included in this study.

**Table 1:** Laboratory parameters

**Preoperative laboratory parameters**	**All**	**Converted n = 14**	**Non-converted n = 9**	**p-value**
aPTT (sec)	26 (24;28)	26.5 (24.8;28)	24 (24;34)	0.557
Creatinin (mg/dl)	0.91 (0.79;1.14)	0.905 (0.81;1.11)	1.0 (0.81;1.24)	0.557
CRP (mg/l)	3.2 (3;15.4)	3.2 (3;20.8)	3 (3;11.8) 0.877	0.877
CK (U/l)	75.5 (63.5;149.8)	77 (64.5;138)	76 (61;192.5)	0.781
CK-MB (U/l)	15 (15;21)	15 (11.5;17)	18 (12;25)	0.516
GOT (U/l)	26 (23;28.8)	25.5 (19.8;28.5)	27 (23;31)	0.557
GPT(U/l)	21 (15;24)	20 (14.5;28)	21 (16;23.5)	0.744
Hemoglobin (g/dl)	13.3 ± 2.1	13.3 ± 2.4	13.5 ± 1.9	0.831
Quick (%)	90.1 ± 20.5	89.6 ± 17.1	89.7 ± 26.8	0.992
Platelets (x1E9/l)	211.5 ± 70.6	207.9 ± 76.2	216.8 ± 69.5	0.780
Troponin-T (ug/l)	0.42 ± 0.82	0.26 ± 0.39	0.77 ± 1.33	0.492
Uric acid (mg/dl)	46.8 ± 17.1	49.6 ± 19.7	44 ± 12.6	0.460
WCC (x1E9/l)	8.5 ± 1.8	8.9 ± 1.6	8.2 ± 2	0.370
Postoperative but before cardioversion				
Ca (mmol/l)	2.1 ± 0.15	2.11 ± 0.16	2.1 ± 0.15	0.729
Creatinin (mg/dl)	1.57 ± 0.97	1.35 ± 0.77	2.0 ± 1.15	0.113
CRP (mg/l)	126 ± 91.5	128.9 ± 99.5	122.7 ± 89.2	0.880
Erythrocytes (x1E12/l)	3.46 ± 0.34	3.5 ± 3.9	3.4 ± 0.2	0.291
Hematocrit (%)	31.21 ± 3.22	31.6 ± 3.5	31.1 ± 2.8	0.742
Hemoglobin (g/dl)	10.15 ± 1.0	10.3 ± 1.1	10.1 ± 0.88	0.741
HbA1c (%)	5.83 ± 0.87	5.8 ± 0.95	5.9 ± 0.77	0.924
MCH (pg)	29.4 ± 1.53	29.0 ± 1.4	29.9 ± 1.7	0.178
MCV (fl)	90.0 ± 4.04	88.9 ± 3.3	91.6 ± 4.9	0.127
Mg (mmol/l)	1.05 ± 0.28	1.11 ± 0.34	0.96 ± 0.19	0.267
NtproBNP (ng/l)	1576 (833;3573)	1463 (721;2772)	3102 (1087;11838)	0.185
Potassium (mmol/l)	4.70 ± 0.64	4.5 ± 0.49	4.9 ± 0.83	0.153
PTC (ug/l)	0.5 (0.2;0.88)	0.6 (0.2;0.75)	0.4 (0.23 ± 3.5)	0.645
Platelets (x1E9/l)	219.3 ± 82.2	214.1 ± 93.5	232.6 ± 68.7	0.616
Sodium (mmol/l)	140.4 ± 3.7	140.1 ± 3.4	141.2 ± 4.3	0.511
TSH (mU/l)	2.37 ± 3.97	2.9 ± 4.9	1.7 ± 1.9	0.501
WCC (x1E9/l)	12.25 ± 5.5	12.12 ± 4.9	12.9 ± 6.7	0.761

aPTT: activated partial thromboplastin time, CK: creatine kinase, CRP: c-reactive protein, GOT:glutamic oxaloacetic transaminase, GPT: glutamate pyruvate transaminase, HbA1c: Glycated haemoglobin, MCH: mean corpuscular haemoglobin, MCV: mean corpuscular volume, NtproBNP: N-terminal prohormone of brain natriuretic peptide, PTC: procalcitonin, TSH:thyroid-stimulating hormone, WCC: white cell counts.

**Table 2:** Baseline characteristics, past medical history and preoperative medication

	**All**	**Converted n = 14**	**Non-converted n = 9**	**p-value**
Age	69.63 ± 6.34	68.21 ± 7.21	71.22 ± 4.58	0.279
CVA (%)	13	21.4	0	0.253
COPD (%)	13	14.3	11.1	1.000
Diabetis mellitus (%)	17.4	14.3	22.2	1.000
Female (%)	26.1	21.4	33.3	0.643
Height (cm)	172.71 ± 8.33	172.64 ± 8.28	172 ± 9	0.862
Hyperlipidemia (%)	43.5	42.9	44.4	1.000
Hypertension (%)	78.3	78.6	77.8	1.000
PAD (%)	8.7	7.1	11.1	1.000
Renal insufficiency or creatinin >1.1 (%)	8.7	7.1	11.1	1.000
Smoker (%)	21.7	28.6	11.1	0.611
Weight (kg)	80.56 ± 13.17	81.24 ± 14.26	79.33 ± 12.87	0.748
ACE-inhibitors(%)	36.4	23.1	55.6	0.187
Aspirin (%)	86.4	84.6	88.9	1.000
Beta-blockers (%)	72.7	69.2	77.8	1.000
Ca-antagonist (%)	36.4	30.8	44.4	0.662
Clopidogrel (%)	4.5	7.7	0	1.000
Cortison (%)	4.5	7.7	0	1.000
Diuretics (%)	31.8	38.5	22.2	0.648
Statin (%)	63.6	69.2	55.6	1.000

COPD: chronic obstructive pulmonary disease,CVA: cerebrovascular accident, PAD: peripheral arterial disease.

**Table 3:** Intraoperative, postoperative and echocardiography data

	**All**	**Converted n = 14**	**Non-converted n = 9**	**p-value**
Cardioplegia type				0.444
Buckberg (%)	40	46.3	28.6	
Calafiore (%)	60	53.8	71.4	
CPB time (min)	109.86 ± 51.3	122.15 ± 59.54	94.13 ± 33.01	0.240
Operation duration (min)	233.52 ± 88.41	242.92 ± 114.4	223.33 ± 38.58	0.628
Operation urgency				0.441
Emergent (%)	4.3	7.1	0.0	
Urgent (%)	13	7.1	22.2	
Elective (%)	82.6	85.7	77.8	
Reperfusion time (min)	36.41 ± 22.08	38 ± 26.32	36.63 ± 14.11	0.894
Total ischemic time (min)	64.05 ± 33.66	71.46 ± 38.27	52.5 ± 25.13	0.230
Hospital stay (days)	16.29 ± 5.72	16.4 ± 6.29	15.67 ± 5.61	0.818
Intensive care unit stay (days)	4 (2.75;6.25)	4 (3;6)	4 (2;19.5)	0.972
IABP	5	0	14.3	0.350
Ventilation duration (in hours)	17.45 ± 8.44	18.94 ± 10.31	49.67 ± 13.32	0.256
Ejection fraction (%)	55.82 ± 9.68	58 ± 8.43	42.33 ± 5.51	0.779
Left atrium (mm)	43.22 ± 7.5	41 ± 6.56	42.33 ± 5.51	0.779
Mitral insufficiency (%)	20	25	0	1.000
Interventricular septum (mm)	11.5 ± 2.59	10.43 ± 2.3	14 ± 1	0.036

CPB: Cardiopulmonary bypass, IABP: Intra-aortic balloon pump.

**Figure 2:**
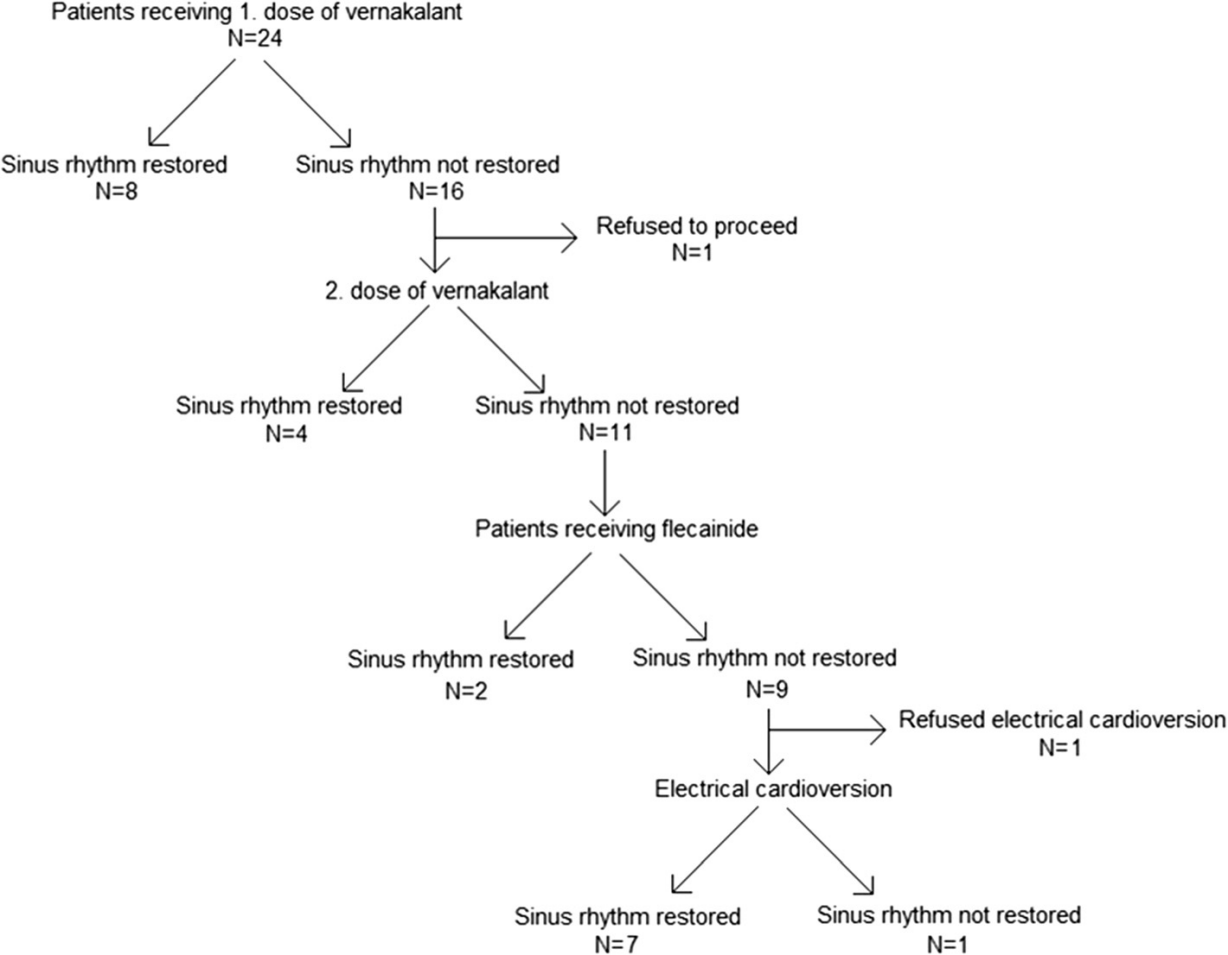
Flowchart of studydesign and success of antiarrhythmic drugs and EC.

The mean age of the population was 69.6 ± 6.3 years and 26.1% of patients were female. 14 patients (60.9%) were successfully converted into a stable sinus rhythm after treatment with vernakalant or combination vernakalant/flecainide. A stable sinus rhythm could be restored in 12 patients (52.2%) after isolated treatment with vernakalant, and in 2 further patients (cumulative 60.9%) after additional dose of flecainide. One patient refused to proceed with pharmacological treatment after the first dose of vernakalant. Another patient refused EC after not successful drug combination treatment (Figure 2). In total, we were able to achieve a stable SR in 21 patients (95.5%) after treatment with both antiarrhythmic drugs and EC.

Comparing converters vs. non-converters after pharmacological treatment, there were no statistically significant differences in terms of height, weight, gender distribution, comorbidities, preoperative medication, left ventricular function and left atrium diameter between both groups (Tables 1, 2 and 3). However, we found that the interventricular septum (IVS) in the non-converted group was significantly thicker compared to the converted group: 14.0 ± 1.00 vs. 10.40 ± 2.59 mm (p = 0.036).

## Discussion

In this study, we investigated for the first time, a graduated scheme therapy of postcardiotomy AF with vernakalant, flecainide and EC. Notably, previous research on AF after cardiac surgery was limited to the use of single therapy with either vernakalant, other antiarrhythmic drugs or/and EC. By comparing converted vs. non-converted patients before EC we found significant difference in the thickness of IVS, which therefore might be a potential negative predictor of successful pharmacological cardioversion.

The negative effect of long term AF has been extensively studied in the previous research [2,16,17]. The incidence of AF in postcardiotomy patients may also depend on the type of surgery. It occurs in 32 - 44% after coronary artery bypass grafting (CABG), 35% after OPCAB, 42% after mitral valve, 49%-74% after aortic valve surgery, and 62% of CABG combined with valve surgery [2,18]. AF after cardiac surgery is associated with longer ICU stay and complications, such as low output syndrome, renal failure and stroke [19-22]. There is also a substantial amount of research, mentioned in the updated Guidelines, associating AF with an increased risk of heart failure and high mortality [23]. These facts enhance the importance of rapid cardioversion in order to avoid further adverse events.

Flecainide is a class Ic antiarrhythmic agent which plays an important role in rhythm control in patients with AF and can be administered both orally and intravenously. Vernakalant is a new alternative intravenous atrial selective antiarrhythmic drug developed for rapid conversion of AF into sinus rhythm [24]. This is a sodium- and potassium-channel blocking agent, which effects all phases (0 – IV) of the atrial action potential (AP) and terminates AF by increasing the atrial effective refractory period (AERP) by blocking the potassium (I_Kur_, I_KACh_, I_Kr_ and I_to_) and sodium currents (I_Na_) in a concentration-, rate- and voltage-dependent manner [25].

The effect of vernakalant on the treatment of recent-onset atrial fibrillation has been widely debated in the previous research [7,26-29]. Also, the negative influence of IVS on the conversion into a sinus rhythm has been already indirectly mentioned in the literature. Furthermore, recent studies reported that the incidence of postoperative AF was higher in patients with aortic valve replacement [2]. This is generally consistent with our results as patients with hypertrophied myocardium and therefore hypertrophied IVS usually have severe aortic stenosis which was treated with surgical aortic valve replacement in our study. Therefore, taking into consideration results of previous research and our study and despite the small patient cohort in the present observation, thickness of the IVS might play a significant role in affecting the success of pharmacological conversion rate.

Analyzing data on pharmacological conversion rate in postcardiotomy patients and patients who had not undergone cardiac surgery, we found out that application of both vernakalant and flecainide as monotherapy was associated with worse conversion rates in postcardiotomy patients. Kowey et al. in his prospective randomized double-blind trial reported on the success rate of conversion with vernakalant into a sinus rhythm in postcardiotomy patients which was 47% compared to the success rate in non-surgical patients of up to 55% [7,26]. While there were no useful data regarding conversion rate of postcardiotomy patients after flecainide treatment, the success rate to this drug in non-cardiac surgery patients ranged between 50–67.5% in the previous literature [30-33]. Not surprisingly, our findings showed a conversion rate after vernakalant monotherapy which was slightly higher however comparable to the study by Kowey et al. and resulted in 52.2% of success. Furthermore, our study showed a significant improvement in the conversion rate into a stable sinus rhythm to 60.9% after additional application of flecainide in patients who did not convert after monotherapy with vernakalant. In interpreting these findings, we assume that the combination of both antiarrhythmic drugs is more effective in converting recent onset atrial fibrillation in carefully selected groups of patients with AF after cardiac surgery. This might be also important in regards to the fact that a substantial number of patients resist EC due to sedation and further fears related to this more demanding procedure. Despite that, our analysis revealed a 91.3% conversion rate into a stable sinus rhythm after treatment with both antiarrhythmics and EC. Taking into consideration the fact that two patients who were not converted in a sinus rhythm after vernakalant and flecainide administration, refused further electric cardioversion attempt, the combined cardioversion rate might have been even higher.

Several authors suggested that the use of flecainide in patients with structural heart defects may contribute to a pro-arrhythmogenic side effects [34,35]. These studies are particularly focused on the risk of ventricular arrhythmia in patients with previous coronary heart disease and myocardial infarction or ischemic cardiomyopathy. Therefore, we excluded patients with ischemic cardiomyopathy and previous myocardial infarction from our observation, as mentioned before. Furthermore, vernakalant with the most common side-effects being dysgeusia, sneezing, paresthesia, nausea, and hypotension appeared to have no side-effects even in combination with flecainide in our study. Also, there were no drug-induced life-threatening ventricular arrhythmias observed after combinational treatment with vernakalant/flecainide. However, it might be related to the relatively low number of patients observed in our investigation.

“Classic” antiarrhythmic drugs, such as amiodaron or Beta-blockers were also shown to be an effective treatment in post cardiotomy patients [36]. Compared to a high conversion rate and quick action of vernakalant shown in our study and previous research, amiodaron treatment for recent-onset AF only resulted in 5% conversion within the first 90 min with similar results in post cardiotomy patients [37]. However, well-designed trials evaluating amiodarone and focused on postoperative medical cardioversion are lacking. Also, treatment with this drug can lead to serious complications such as non-cardiac toxicity including pulmonary, hepatic, thyroid, and neurologic side-effects. Moreover, its intravenous administration can be associated with hypotension, bradycardia, and thrombophlebitis, and this agent should be used with caution in patients with severe pulmonary disease and low pulmonary resistance. In contrast, vernakalant is the first marketed drug with relative atrial selectivity and low rate of adverse events reported, indicating a higher safety profile. Beta-blockers are also relatively safe in terms of adverse events, however they are mainly used as the main agents for ventricular rate control rather than for rhythm control [36].

The result of our study may be interpreted to indicate that the oral application of 300 mg flecainide added to intravenously administered vernakalant in patients who did not convert into a sinus rhythm may not only increase the cardioversion rate but also prevent pro-arrhytmogenity and reduce side effects of vernakalant. Recent studies showed that the oral application of a single flecainide dose of 300 mg in patients with structural heart disease is safe and effective in carefully selected patients [2,38].

Our study has several limitations. The main limitation is its non-randomized design and analysis of limited number of patients from a single institution. Furthermore, previous usual therapy strategies with non-class III antiarrhythmics, such as Beta-blockers, as well as electrolytes were not quantified. Larger prospective randomized studies with this aggressive therapy protocol are needed to confirm our preliminary results.

## Conclusion

Our results show that the sequential combinational treatment with vernakalant, flecainide and EC is a recommendable and effective therapy strategy to convert rapidly new onset AF in carefully selected patients after cardiac surgery.

## Abbreviations

AERP: Atrial effective refractory period; AF: Atrial fibrillation; AP: Action potential; CABG: Coronary artery bypass grafting; EC: Electric cardioversion; IVS: Interventricular septum; SR: Sinus rhythm.

## Competing interests

The authors report no competing interests.

## Author’s contributions

MZ, AS and JF participated in the study design, recruited patients, analysed the data and drafted the manuscript. HR, AFP, LK and PBR participated in the study design, data analysis and study coordination. TW, LK, YHC, JW and TW participated in the design of the study and supervised the trial process. All authors read and approved the final manuscript.

## Author’s information

Mohamed Zeriouh, Anton Sabashnikov and Yeong-Hoon Choi share the first/senior author designation.
